# Tissue-like Fracture Toughness and Stress–Relaxation Ability in PVA-Agar-Based Hydrogels for Biomedical Applications

**DOI:** 10.3390/gels11090747

**Published:** 2025-09-17

**Authors:** Ismael Lamas, Bhuvana L. Chandrashekar, Claudia C. Biguetti, Mohammad R. Islam

**Affiliations:** 1 Department of Mechanical Engineering, University of Texas Rio Grande Valley, Edinburg, TX 78539, USA; 2 Biomedical Engineering Program, University of Texas Rio Grande Valley, Edinburg, TX 78539, USA; 3 School of Podiatric Medicine, University of Texas Rio Grande Valley, Edinburg, TX 78539, USA; 4 Materials Science and Engineering Program, University of Texas Rio Grande Valley, Edinburg, TX 78539, USA

**Keywords:** hydrogel, fracture toughness, stress–relaxation, viscoelasticity, double network, PVA-agar

## Abstract

Soft tissues exhibit remarkable stretchability, fracture toughness, and stress–relaxation ability. They possess a large water content to support cellular processes. Mimicking such a combination of mechanical and physical properties in hydrogels is important for tissue engineering applications but remains challenging. This work aims to develop a hydrogel that can combine excellent mechanical properties with cellular viability. The research focused on polyvinyl alcohol (PVA)/agar double-network (DN) hydrogels, fabricated by thermal gelation and freeze–thawing methods. Their mechanical properties were characterized through tension, compression, fracture, and stress–relaxation tests, and their cellular viability was measured through cytotoxicity tests. The results show that the PVA/agar DN gels are highly stretchable (>200%) and compressible (>30%) while containing high water content. The incorporation of agar by 6 wt% improved the fracture toughness of hydrogels from 1 to 1.76 kJ/m^2^. The degree of stress–relaxation, a key indicator of gel viscoelastic properties, improved by roughly 170% with an increase in agar content from 0 to 6 wt%. Cytotoxicity analysis showed that the gels, being physically cross-linked, were able to promote cellular proliferation. This work shows that tough and viscoelastic PVA/agar DN gels are suitable for soft tissue engineering applications, especially cartilage repair.

## 1. Introduction

Hydrogels are promising biomaterials as artificial scaffolds for damaged tissue repair and regeneration. They closely mimic the physical attributes of biological tissues but are generally inferior in mechanical properties [1]. Soft tissues (e.g., articular cartilage, aorta, and skin) exhibit high fracture toughness to resist crack propagation [2,3,4] as well as excellent stress–restress–relaxation ability to dissipate energy during deformation [5,6,7]. Hydrogel scaffolds must closely recapitulate these mechanical properties to support physiological loads and in vitro tissue growth [8,9]. More importantly, they must achieve these mechanical properties with high water content and biocompatibility to support cellular proliferation [10]. Hydrogels that meet both the mechanical and physical needs of tissue scaffolds are essential in the tissue engineering field [11,12,13,14].

Recent research efforts have introduced a large set of strong and tough hydrogels, primarily using the double-network (DN) strategy [15,16,17]. These hydrogels combine two complementary polymer networks, where a brittle network provides an effective toughening mechanism through permanent rupture, and a ductile network preserves the gel integrity [18,19]. Consequently, DN hydrogels achieve remarkable fracture toughness (0.1–10 kJ/m^2^) [19,20,21,22], comparable to or higher than most tissues. Improvement in hydrogel mechanical properties has also been demonstrated by other mechanisms, including but not limited to fiber reinforcement [23,24], particle filling [25,26], and triple network system [27,28]. However, these advanced hydrogels do not address other mechanical requirements of artificial scaffolds, especially stress–relaxation ability. This is primarily due to the presence of one or both chemically cross-linked polymer networks. Chemical cross-linking reduces chain mobility in the polymer network by forming permanent covalent bonds between polymer chains. It renders the resultant hydrogel highly elastic with little or no stress–relaxation ability [29,30]. Stress relaxation is a characteristic property of soft tissues, where the stress (internal resistance) within the tissue decreases gradually over time when it is held at a constant strain. It is a key regulator of cellular processes. It primarily originates from the viscoelasticity of tissue extracellular matrix (ECM) constituents (e.g., collagen) [7]. Viscoelastic relaxation involves the dynamic reorganization of the collagen fiber network, providing crucial biophysical signals to cells [7,31]. DN hydrogels are rarely designed for viscoelastic properties, although the impact of hydrogel viscoelasticity on cell mechanobiology is well-established [32]. The presence of chemical cross-linking also makes chemical gels cytotoxic, which is also not desirable for tissue scaffolds. In contrast to chemical gels, physical hydrogels (e.g., agar and alginate) can undergo rapid stress–relaxation like biological tissues [29]. This is because physical gels are formed through chain entanglement (agar) or non-covalent ionic bonds (alginate), which allows chain mobility and reorganization of the polymer network. However, most physical hydrogels are inherently fragile with small fracture toughness (0.001–0.01 kJ/m^2^) [33]. Hence, advanced hydrogel biomaterials that can combine the large fracture toughness of chemical DN gels with the strong stress–relaxation ability of physical gels are essential.

Within this motivation, this work investigates a physically cross-linked DN hydrogel consisting of agar and polyvinyl alcohol (PVA). PVA-agar hydrogels are fabricated by combining thermal gelation (agar) with the freeze–thawing method (PVA). The advantage of this fabrication approach is that it does not involve any toxic chemical component and, therefore, the hydrogels are inherently non-toxic. The disadvantage is that the fabrication is time-consuming, which typically involves several days [34]. PVA-agar DN gels have been studied in prior work to demonstrate their useful properties for multifunctional applications [35,36]. Mohammad et al. showed that the addition of agar into PVA hydrogel leads to a high elastic modulus and rapid self-healing ability at room temperature [37]. The combination of PVA and agar can form a conductive hydrogel when soaked in ammonium sulfate [38]. Microstructural analysis of PVA/agar gels through scanning electron microscopy (SEM) imaging showed that agar also acts as a pore-inducing agent to provide the PVA-agar hydrogels with a macroporous structure [39]. The chemical structure of PVA/agar gels has also been studied in the literature [37,38,39]. These studies showed that PVA-agar DN hydrogels provide several attractive properties for tissue engineering applications, especially self-healing ability, macroporosity, and conductivity. However, the fracture mechanics and stress–relaxation behavior of PVA-agar hydrogels have not been characterized previously, which is the major novelty of this work. This work presents, for the first time, a systematic characterization of fracture toughness and stress–relaxation behavior of PVA-agar hydrogels, in addition to their mechanical behavior under tension and compression. The swelling behavior and cytotoxicity of the gels have also been characterized to demonstrate the versatility of PVA-agar hydrogels for tissue engineering applications.

## 2. Results and Discussion

### 2.1. Swelling Behavior

**Figure 1:**
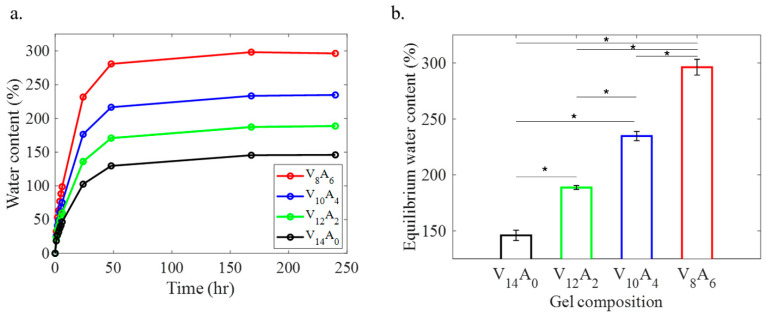
Swelling behavior of PVA-agar composite hydrogels. (**a**) Variation of water content in the hydrogels over ten days (240 h) and (**b**) equilibrium water content of the hydrogels. The symbols (*) in (**b**) denote statistical significance (*p* < 0.05).

The swelling of the gels was monitored by measuring the weight of the gel from the dry state to the fully swollen state for ten days (Figure 1a). Both PVA and PVA-agar gels demonstrated similar swelling dynamics, where rapid swelling occurred within the first 6 h, and all the gels reached equilibrium within 48 h. The incorporation of agar enhanced the swelling of the composite gels more than two times that of the pure PVA gel. With an increase in the agar content from 0 to 6 wt%, the equilibrium water content of the composite gels increased from 146 to 296 wt%. The swelling properties of the gels were calculated with respect to their dry weights.

### 2.2. Mechanical Properties

#### 2.2.1. Tension and Compression Behavior

**Figure 2:**
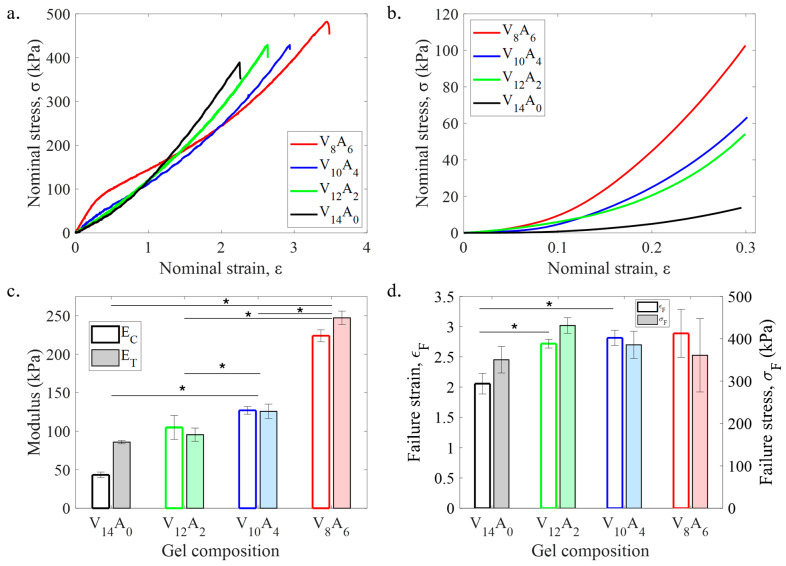
Mechanical behavior of PVA-agar composite hydrogels in tension and compression. Representative (**a**) tensile and (**b**) compressive stress–strain curves of four hydrogels with different compositions. Variation of (**c**) elastic modulus in tension (E_T_) and compression (E_C_), and (**d**) tensile failure strength ($\sigma_{F}$) and strain ($\epsilon_{F}$) among four hydrogels with increasing agar content. Error bars in (**c**,**d**) represent standard deviation among three samples. The symbols (*) in (**c**,**d**) denote statistical significance (*p* < 0.05).

The mechanical synergy of the PVA-agar DN gel was evaluated in uniaxial tension and compression (Figure 2). In uniaxial tension, the dog-bone-shaped gel samples were stretched to rupture. Figure 2a shows the tensile stress–strain responses of four gels with different compositions. The pure PVA gel (V_14_A_0_) shows a slightly nonlinear response with no yielding or stiffness degradation before failure. The incorporation of agar leads to a stiffer response for the composite hydrogels at the small strain regime (ϵ≤1), where the effect is the strongest for the hydrogel with 6 wt% agar (V_8_A_6_). In the presence of high agar content, the gels (V_8_A_6_ and V_12_A_2_) exhibit yielding at around ϵ ≈ 0.4. The large strain response of composite gels is softer than pure PVA gels, which is associated with the breakage of the agar network.

Figure 2b illustrates the compressive stress–strain curves of four gels with different compositions. Similar to the tensile response, the composite gel demonstrated a stiffer response in compression when compared to the pure PVA gel. Both PVA and the PVA-agar composite gels could sustain large compression (ϵ≥ 0.8) without failure. It is interesting since the pure agar gel fails within 30% compressive strain [36].

To understand the effect of agar content, the mechanical properties of four gels were calculated from the stress–strain curves (Figure 2c,d). The addition of agar significantly enhanced the elastic modulus of the composite gels (Figure 2c). As the agar content was varied from 0 to 6 wt%, the elastic modulus (E) of the composite gel increased by roughly three times that of pure PVA gel. The compressive modulus also gradually increased from 50 kPa to 225 kPa for 6 wt% agar content. Tensile samples of pure PVA gels failed around ϵ≈2, whereas PVA-agar gels failed at ϵ≈ 2.5 to 2.8. Tensile failure stress of the gels was around 380–420 kPa, irrespective of agar content.

#### 2.2.2. Fracture Behavior

**Figure 3:**
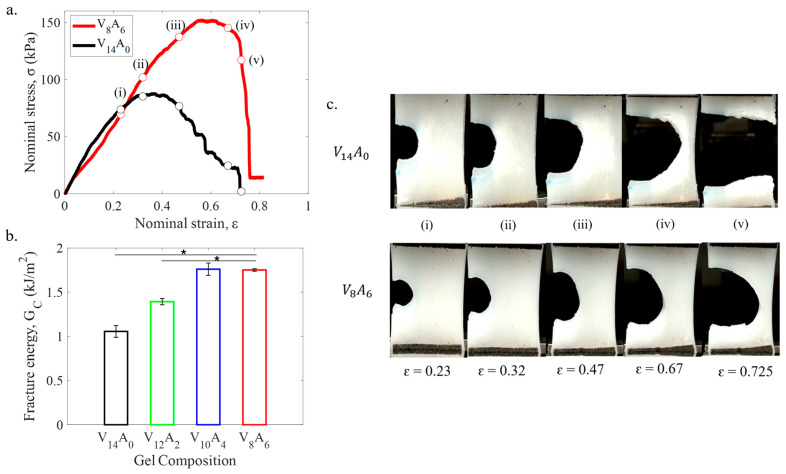
Fracture behavior of the PVA-agar gels. (**a**) Stress–strain curves of V_14_A_0_ and V_8_A_6_ gels, as measured with 15 mm notched specimens under pure shear configuration. (**b**) Variation of fracture energy (G_C_) among different hydrogels with increasing agar content. Error bars represent standard deviation among three samples. (**c**) Crack propagation snapshots of V_14_A_0_ (top row) and V_8_A_6_ (bottom row) gel specimens with 15 mm notch at various strain levels. The symbols (i–v) in the stress–strain curves of (**a**) correspond to the snapshots in (**c**). The symbols (*) in (**b**) denote statistical significance (*p* < 0.05).

The fracture energies of the PVA-agar gels were measured by pure shear tests. Figure 3a shows the representative stress–strain curves of two notched hydrogel specimens. The PVA-agar composite gel (V_8_A_6_) also exhibited a stiffer response compared to pure PVA gel (V_14_A_0_). It indicates larger forces are required to propagate the crack through the composite gel compared to pure PVA gel. Figure 3b illustrates the variation of critical fracture energy ($G_{C}$) among different hydrogels with increasing agar content. $G_{C}$ increased linearly from 1.06 kJ/m^2^ to 1.76 kJ/m^2^ as the agar content was increased from 0 to 4 wt%. The fracture energy did not improve with a further increase in agar content to 6 wt%.

To better understand the fracture process in the PVA-agar gels, the crack propagation in the notched gel specimens was monitored as a function of strain (Figure 3c). The snapshots in Figure 3c correspond to the symbols in the stress–strain curves of Figure 3a. The pure PVA gel (V_14_A_0_) demonstrated crack blunting behavior up to ϵ ≈ 0.4. The crack initiated advancing for ϵ> 0.43, and it transformed into a running crack with increasing strain. In contrast, the PVA-agar composite gel (V_8_A_6_) showed stable crack blunting behavior with semicircular crack shape up to ϵ ≈ 0.6. The rapid crack propagation initiated at higher strain (ϵ> 0.65) in the composite gel. This work systematically characterized for the first time the fracture behavior of PVA-agar gels.

#### 2.2.3. Stress–Relaxation Behavior

**Figure 4:**
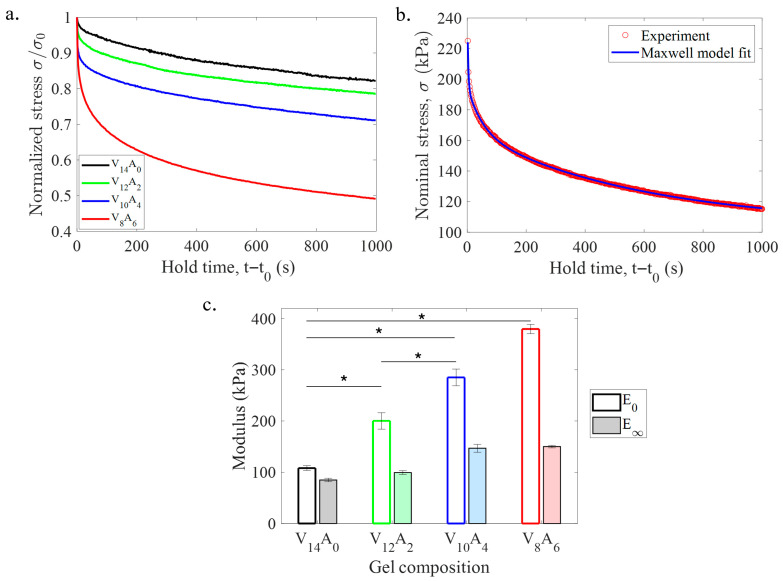
Stress–relaxation behavior of PVA-agar gels, as measured by compression testing. (**a**) Normalized stress–relaxation curves of the gels with various combinations of PVA and agar. The stress (σ) is normalized by the peak stress ($\sigma_{0}$) at the maximum strain (ϵ=0.1) for comparison. (**b**) Representative viscoelastic (generalized Maxwell) model fit for a PVA-agar composite gel (V_8_A_6_). (**c**) Variation of instantaneous ($E_{0}$) and equilibrium ($E_{\infty }$) moduli with agar content. Error bars in (**c**) represent standard deviation among three samples. The symbols (*) in (**c**) denote statistical significance (*p* < 0.05).

The time-dependent stress–relaxation behavior of the PVA-agar gels is shown in Figure 4a. The vertical axis is normalized by the maximum stress ($\sigma_{0}$) for direct comparison of the gels. The pure PVA gel (V_14_A_0_) showed weak stress–relaxation, such that the stress dropped by roughly 18% of $\sigma_{0}$ over 1000 s. In contrast, the PVA-agar composite gels showed improved stress–relaxation ability with an increase in agar content. With the increase in agar content from 0 to 6 wt%, the degree of stress–relaxation increased from 18 to 50% over 1000 s. The effect is associated with the viscoelasticity of agar, which allows agar gels to undergo strong stress–relaxation (~80%) on a similar timescale [29].

The stress–relaxation curves of the PVA-agar gels were analyzed using the generalized Maxwell model. The model effectively represented the dominant relaxation behavior of the hydrogels at different timescales with a high goodness of fit (R^2^ > 0.9) for all cases. A representative model fitting the stress–relaxation curve of a PVA-agar gel is shown in Figure 4b. The model prediction indicates that the hydrogels undergo rapid relaxation at the initial stage (up to 100 s), and the rate of relaxation decreases gradually as the gels approach the equilibrium limit. Figure 4c shows the variation of instantaneous ($E_{0}$) and equilibrium ($E_{\infty }$) moduli of the gels among different hydrogels with increasing agar content. Both moduli increased gradually with the increase in agar content, like elastic modulus. The viscoelastic ratio ($E_{\infty }/E_{0}$) also increased from 0.78 to 0.39, indicating the PVA-agar hydrogels became more viscous with the increase in agar content. This is the first study that systematically characterized the stress–relaxation behavior of PVA-agar hydrogels.

### 2.3. Cytotoxicity

**Figure 5:**
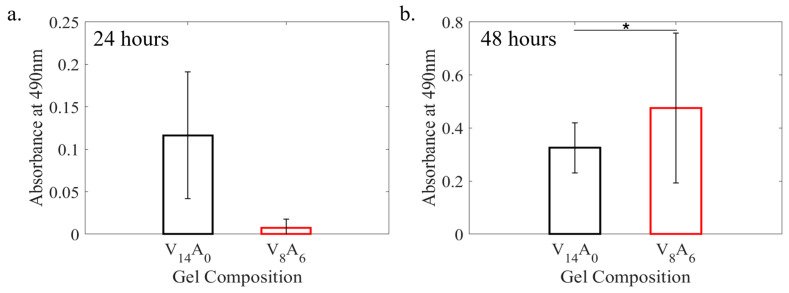
MTS assay showing the metabolic activity of human gingival fibroblast (HGF-1) cells in pure PVA (V_14_A_0_) and PVA-agar (V_8_A_6_) hydrogels after (**a**) 24 and (**b**) 48 h. The results are presented as mean absorbance values with standard deviations. The symbol (*) in (**b**) denotes statistical significance (*p* < 0.05).

The pure PVA (V_14_A_0_) and PVA-agar (V_8_A_6_) gels were tested for their cytotoxicity using an MTS-based assay, as shown in Figure 5. The results indicate that the metabolic activity increased by roughly 109% from 24 to 48 h of cell culture for the pure PVA gel. The metabolic activity increased by more than 20 times for the PVA-agar composite gel. The increase in metabolic activity suggests that cells are viable within the hydrogel environments, where the biocompatibility effect is enhanced by the presence of agar in the hydrogel.

### 2.4. Discussion

Soft biological tissues exhibit large fracture toughness and strong stress–relaxation ability, which not only safeguard them against mechanical damage but also support cellular growth. It is well-established that chemically cross-linked DN hydrogels can provide tissue-like fracture toughness, but they have poor stress–relaxation ability. The chemical cross-linking also affects the biocompatibility of these hydrogels. In this work, PVA-agar DN hydrogels have been studied as a candidate for soft tissue replacement. The DN gel consists of an entangled network of agar double helices as the first system and a semi-crystalline PVA network as the second one. While PVA-agar hydrogels have been studied in prior studies, their fracture toughness and stress–relaxation properties remain unexplored. This work systematically characterized these two properties to demonstrate a simple approach to achieve tissue-like large fracture toughness and stress–relaxation ability in PVA-agar hydrogels without chemical cross-linking.

The elastic modulus of the PVA-agar gels increased significantly with the agar content. For 6 wt% agar content, the tensile modulus of V_8_A_6_ gel increased by 190% compared to V_14_A_0_ gel. Interestingly, the pure PVA gel showed a larger tension–compression asymmetry in terms of elastic modulus compared to the PVA-agar composite gels. The asymmetry is associated with the formation of crystalline zones in the PVA hydrogels, which can make the gel structure anisotropic. The presence of agar reduces the anisotropy of the gel structure. Unlike elastic modulus, tensile strength (*σ_F_*) was not significantly affected by the increment in agar content. The relatively weak effect on tensile strength is intuitive since pure agar gels are significantly weak in tension with a kPa range tensile strength [36]. It confirms that the tensile behavior of the gels is largely dominated by the PVA component. In compression, the DN gels did not undergo any damage or fracture even at 30% compression (Figure 3b). The ability to sustain large compression is essential for hydrogel biomaterials to replace connective tissues like cartilage [40].

The effect of agar is significant on the fracture toughness of the PVA-agar DN gels. The fracture toughness of the PVA-agar DN gel increased from 1.06 to 1.76 kJ/m^2^ for 6 wt% agar content. While the fracture toughness values of PVA-agar DN gels represent the lower limit of most DN gels, they are comparable to several biological tissues. For example, articular cartilage has a fracture toughness of 0.5–2 kJ/m^2^ [2,3], and the fracture toughness of the aorta is around 0.3–0.5 kJ/m^2^ [4]. Interestingly, the fracture toughness did not improve monotonically as the agar content was increased from 4 wt% to 6 wt%. The pure agar hydrogel is a brittle hydrogel with a low fracture toughness of approximately 0.01 kJ/m^2^ [33]. For a relatively large weight fraction (42% for 6 wt%) of agar, the PVA/agar DN gel can become weak with no enhancement in fracture toughness. It suggests that agar contributes to the DN gel’s fracture toughness by its viscoelastic stress–relaxation ability, not by its ability to resist crack propagation.

The stress–relaxation ability of PVA-agar DN gels increases gradually with an increase in the agar content. The fast load relaxation over the short timescale (100 s) is dominated by the topological kinematics of the polymer chains and reversible bonds (viscoelastic relaxation). The stress–relaxation ability of PVA-agar gels is comparable to several biological tissues. In this work, V_8_A_6_ gel demonstrates a 50% stress–relaxation. In a similar timescale, biological tissues like the brain, heart, and skin exhibit 50–60% stress–relaxation [7]. Joint tissues like the cartilage and meniscus exhibit 80–90% stress–relaxation on a similar timescale [41,42]. In contrast, chemical hydrogels like polyacrylamide show less than 10% stress–relaxation on a similar timescale [29]. While stress–relaxation data are not available for most chemical DN hydrogels in the literature, it can be inferred that stress–relaxation behavior of chemical DN gels will be on the same order as polyacrylamide due to the inherent permanently cross-linked structure [30].

The cytotoxicity results indicate that the incorporation of agar into the PVA hydrogel induces higher metabolic activity of fibroblasts than the pure PVA hydrogel. This also indicates greater cellular proliferation within the PVA-agar hydrogel environment. The effect is likely associated with the enhanced stress–relaxation ability of the PVA-agar hydrogel. Prior studies showed that enhanced stress–relaxation significantly affects cellular migration and proliferation [31]. While the cytotoxicity tests were performed for pure PVA and one DN gel (V_8_A_6_), it is expected that other DN gels will also show similar behavior because the PVA/agar DN gels of this work have been developed based on a physical cross-linking mechanism without the application of toxic chemicals.

The present study on PVA/agar gels has limitations that must be considered when interpreting the results, and that require further investigation. First, the swelling behavior of PVA/agar gels was evaluated under deionized water with a neutral pH level. For practical applications, it is important to study gel swelling under different pH levels to better mimic their properties under physiological conditions. However, the focus of this work is on hydrogel mechanical properties, especially how to achieve tissue-like viscoelastic and fracture properties in hydrogel biomaterials. The swelling tests were performed to measure the gel mechanical properties under fully swollen conditions. A future study will comprehensively characterize the swelling behavior of PVA/agar gels under different pH levels.

Second, this study primarily focuses on the mechanical properties of PVA/agar gels, but other properties of the gels, including but not limited to antioxidant, anti-inflammatory, and antibacterial properties, must also be studied for clinical applications. A comprehensive characterization of these properties for PVA/agar gels requires a detailed study, which is beyond the scope of the current study. Third, the cytotoxicity of PVA/agar gels was measured for a limited time. The main purpose of cytotoxicity tests in this work is to demonstrate how the mechanical properties (e.g., viscoelasticity) can be leveraged to modulate cellular interaction with the biomaterial. A future study will focus on a comprehensive analysis of the cytotoxicity of PVA/agar gels for different applications. Finally, a systematic analysis of the structure–property relationships of PVA-agar gels is crucial to obtain insights about their material behavior. However, it requires additional study, which is beyond the scope of the current work. Future studies will focus on correlating surface and microstructural features with the mechanical properties of PVA-agar gels.

This work demonstrates that the mechanical properties, especially fracture toughness and stress–relaxation ability of PVA hydrogels, can be significantly improved based on a single parameter, which is agar content. Toughening of PVA-agar DN gels involves energy dissipation like conventional DN gels. However, one important distinction is that energy dissipation in PVA-agar gels is largely viscoelastic, involving rapid association and dissociation of the physical bonds, in contrast to the permanent breakage of covalent bonds. The fundamental design strategy of most DN gels is to sacrifice the strong network to achieve large fracture toughness and use the second soft and ductile network to preserve structural integrity [43]. An opposite design strategy is employed in this work, where the weak network (agar) is sacrificed, and the strong PVA network is retained to achieve large fracture toughness. In summary, the PVA-agar hydrogels provide a useful combination of mechanical and physical properties for load-bearing tissue engineering applications.

## 3. Conclusions

This work presents the synthesis and characterization of a fully physically cross-linked DN hydrogel by combining PVA and agar for tissue engineering applications. The major achievements of the work include obtaining an effective combination of mechanical properties for the PVA-agar DN gels and ensuring cellular viability. PVA-agar gels exhibit good stiffness (0.1–0.2 MPa), moderate strength (0.3–0.4 MPa), and high fracture toughness (1–1.76 kJ/m^2^), comparable to soft tissues. Different from existing DN gels with one or both covalent networks, this work showed that the PVA-agar DN gels are highly viscoelastic with strong stress–relaxation ability, like various soft tissues. The agar content also induces higher cellular metabolic activity, enhancing the biocompatibility of PVA-agar DN gels. Overall, these promising characteristics demonstrate the potential of PVA-agar gels for clinical studies on the repair and regeneration of load-bearing tissues. Future studies will focus on the in vivo assessment of PVA/agar gels for tissue engineering applications (e.g., damaged cartilage replacement and wound healing) to understand how the tissue-mimetic mechanical properties of PVA-agar gels facilitate their healing ability and integration with native tissue, reducing the risk of rejection and undesired immune responses.

## 4. Materials and Methods

### 4.1. Hydrogel Fabrication

**Figure 6:**
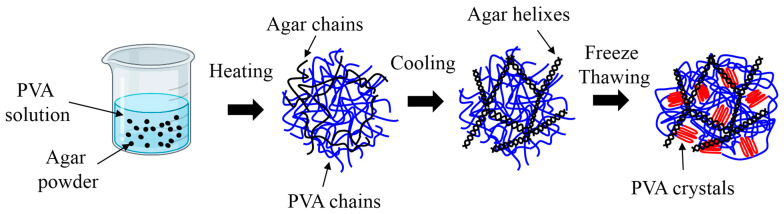
Schematic illustration of the PVA-agar gel formation process. Agar molecules relax into straight chains upon heating beyond its melting temperature. Subsequent cooling to room temperature produces double-helical bundles, which form an interconnected polymer network. The freeze–thawing cycles produces the PVA network cross-linked by the crystallization regions.

PVA-agar DN hydrogels were fabricated based on a straightforward protocol similar to prior work [37,38]. Agar and polyvinyl alcohol (PVA; 146,000–186,000 g/mol and 99% hydrolyzed) were obtained from Sigma-Aldrich Inc. (Raleigh, NC, USA). First, a solution of PVA was prepared by mixing solid PVA powder with deionized water at 85 °C. Next, agar powder was added to the PVA solution, and the mixture was stirred further to obtain a homogeneous solution. The PVA-agar mixture was placed in 3D-printed molds with custom shapes. The molds were cooled down to room temperature, which transforms agar chains into double-helical bundles, and the bundles form the entangled agar network [44]. Subsequently, the molds were covered with parafilm and subjected to three freeze–thaw cycles (freezing at −20 °C for 18 h and thawing at room temperature for 6 h). The freeze–thawing cycles form the PVA network with crystalline regions [35]. It results in a physically cross-linked DN hydrogel consisting of two hydrogen-bonded polymer networks with helical (agar) and crystalline (PVA) architectures (Figure 6). All gels were swollen in deionized (DI) water and stored in a refrigerator (4 °C) before mechanical testing.

The total polymer content of PVA-agar DN hydrogels was kept constant at 14 wt% for all cases. The PVA content was varied as 14, 12, 10, and 8 wt%, whereas the agar content was varied as 0, 2, 4, and 6 wt% of DI water. For the brevity of representation, the PVA-agar gels are referred to as V_x_A_y_ throughout this article. Here, ‘V’ and ‘A’ symbolize PVA and agar, respectively. The subscripts (x and y) indicate the weight percentiles of the solid polymer. For example, V_8_A_6_ represents a DN hydrogel of 8 wt% PVA and 6 wt% agar.

### 4.2. Swelling Study

To study the swelling behavior, the hydrogels were dried at room temperature for a week. The dried gels were placed in deionized water to completely re-swell them. The complete swelling of the gels took approximately two days (48 h). The mass of the gel samples was measured periodically during the swelling process. The water content was calculated as the percentile change in the mass of wet gel with respect to that of the dry gel.

### 4.3. Mechanical Testing

#### 4.3.1. Compression and Tension Tests

**Figure 7:**
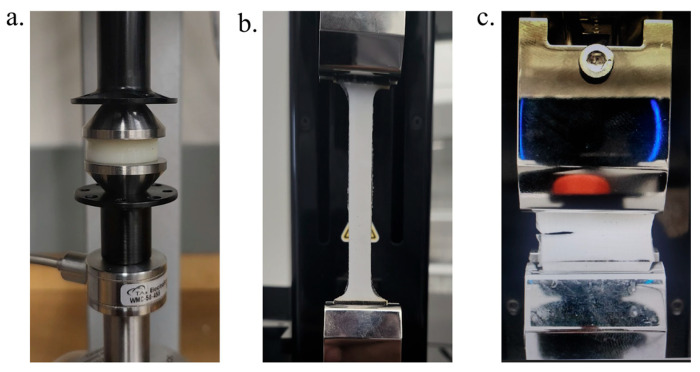
Mechanical testing configurations of PVA-agar hydrogels—(**a**) cylindrical, (**b**) dog-bone-shaped, and (**c**) rectangular notched specimens, as used for uniaxial compression and stress–relaxation, tension, and fracture tests, respectively.

Cylindrical specimens with 20 mm diameter and 10 mm height were used for compression testing (Figure 7a). For uniaxial tension tests, dog-bone-shaped specimens (L_g_ = 50 mm, W_g_ = 10 mm, and t_g_ = 2 mm) were used (Figure 7b). The compression tests were performed on an Electroforce 5500 low-force mechanical tester (TA instruments, New Castle, DE, USA) with a 200 N load cell. The tension tests were performed using a Univert mechanical tester (CellScale, Waterloo, ON, Canada) with a 20 N load cell. All the tests were performed under displacement control with a fixed rate of 30 mm/min. All the tests were performed in the air with fully swollen hydrogel samples. For the compression tests, the cylindrical samples were subjected to a maximum strain of 30%. The tensile specimens were stretched until the rupture. The nominal stress (σ) was calculated as the force divided by the cross-section area of the undeformed samples. The nominal strain (ϵ) was calculated as the ratio of the change in specimen length and the undeformed length of the specimen. The failure strength ($\sigma_{F}$) was reported as the peak stress from the tensile stress–strain curves. The failure strain ($\epsilon_{F}$) was defined as the strain level corresponding to the peak stress in the tensile stress–strain curve.

#### 4.3.2. Fracture Tests

Fracture tests were performed with rectangular specimens (30 mm × 25 mm × 5 mm), as shown in Figure 7c. The specimens were glued between two plastic sheets (10 mm height) for gripping the specimens in pure shear fracture test configurations [45]. Both notched (10 mm notch length) and unnotched samples were tested for fracture tests. To calculate the fracture toughness (*G*_c_), the notched specimen was stretched until rapid and unstable crack propagation occurred at the critical strain ($\epsilon_{c}$). *G*_c_ was then calculated as U($\epsilon_{c}$)w, where U is the strain energy up to a strain level of $\epsilon_{c}$, and w is the width of the specimen (w = 25 mm). An unnotched specimen was stretched to the same $\epsilon_{c}$, and the area under the stress–stretch curve was used as U($\epsilon_{c}$). The fracture tests were performed on the same mechanical testing machine as the tension tests.

#### 4.3.3. Stress–Relaxation Tests

The stress–relaxation tests were performed using a compression-based protocol. Cylindrical specimens (20 mm diameter and 10 mm height) were used, similar to compression test specimens (Figure 7a). For each relaxation test, the compression platen was programmed through the machine software to compress the gel to a depth of 1 mm (ϵ=0.1) within a ramp time of 2 s. After the compression step, the platen was held at the fixed position, and the decay of the force P(t) was captured for 1000 s to characterize the relaxation behavior of the gels. The force data were converted to stress (σ) data by dividing the force by the cross-section area (A) of the sample.

The viscoelastic properties were calculated based on the generalized Maxwell model [42,46], consisting of a linear spring connected in parallel with *n* number of Maxwell units (series connection of a linear spring and a dashpot). The model uses a series of exponentials to define the relaxation modulus function, E(t):

(1) $$E\left(t\right)=E^{*}+\sum_{n}E_{n}\exp\left(-\frac{t}{\tau_{n}}\right),$$

where $E^{*}$ is the elastic modulus of the linear spring, $E_{n}$ is the modulus of the *n^th^* Maxwell element, and $\tau_{n}$ is the characteristic relaxation time of the $n^{th}$ Maxwell element. The instantaneous modulus ($E_{0}$) is defined as $E_{0}=E^{*}+\sum_{n}E_{n}$ and the equilibrium modulus ($E_{\infty }$) is calculated as $E_{\infty }=E^{*}$. The ratio of two moduli ($E_{\infty }/E_{0}$) is called the viscoelastic ratio. The corresponding stress can be calculated as:

(2) $$\sigma \left(t\right)=\frac{P\left(t\right)}{A}=E\left(t\right)\epsilon_{0}=\left[E^{*}+\sum_{n}E_{n}\exp\left(-\frac{t}{\tau_{n}}\right)\right]\epsilon_{0}\;$$

Equation (2) was directly fitted to the experimental stress–relaxation curves by using a least square optimization algorithm for *n* = 3, as developed in our previous work [5].

### 4.4. Cytotoxicity Test

Human gingival fibroblasts (HGF-1) were obtained from the American Type Culture Collection in Manassas, VA, USA. They were cultured in T-75 flasks. The cells reached 70–80% confluency at 37 °C in an atmosphere of 5% CO_2_. The culture medium used was Dulbecco’s Modified Eagle Medium (DMEM). It was supplemented with 10% Fetal Bovine Serum (FBS) and 1% penicillin–streptomycin (10,000 U/mL). For the experiments, hydrogels (V_14_A_0_ and V_8_A_6_) were manufactured under sterile conditions in 48-well plates. Cells were seeded at a density of 8 × 10^4^ cells per well either on the hydrogels or directly on the plate dishes as negative controls (NCs). Four technical replicates were allocated for each group. Cell viability was assessed using the MTS assay (Promega, Madison, WI, USA) to measure metabolic activity via tetrazolium reduction at 24 and 48 h post-seeding. A total of 100 µL of reagent was added per 500 µL of media. Cells were incubated for 4 h at 37 °C. After the incubation time, 200 µL of the supernatant from each well was transferred from each well to a new 96-well plate, giving at least 3 replicates per group. The absorbance was measured at 490 nm to provide quantitative data. Blanks for the hydrogels were also performed as internal controls to check for possible bacterial contamination or variations in readings due to the MTS reacting with the hydrogel itself. No bacterial contamination was detected, nor were there differences in the MTS blank readings between the hydrogel and the plate.

### 4.5. Statistical Analysis

The swelling, mechanical, and cytotoxicity tests were repeated for multiple replicas of each PVA/agar gel. The results are presented as mean values with standard deviations to highlight their representative properties and property variation across multiple samples. Mean differences of the hydrogel properties were analyzed using one-way analysis of variance (ANOVA) and post hoc pairwise multiple comparison tests (Bonferroni method), similar to our prior work [5]. Differences were considered significant for *p* < 0.05. Statistical analysis was performed using MATLAB (version R2023a, statistics and machine learning toolbox).

## Data Availability

The data will be available from the corresponding authors upon reasonable request.

## References

[1] Oyen M.L. (2014). Mechanical characterisation of hydrogel materials. Int. Mater. Rev..

[2] Simha N., Carlson C., Lewis J. (2004). Evaluation of fracture toughness of cartilage by micropenetration. J. Mater. Sci. Mater. Med..

[3] Taylor D., O’Mara N., Ryan E., Takaza M., Simms C. (2012). The fracture toughness of soft tissues. J. Mech. Behav. Biomed. Mater..

[4] Chu B., Gaillard E., Mongrain R., Reiter S., Tardif J.-C. (2013). Characterization of fracture toughness exhaustion in pig aorta. J. Mech. Behav. Biomed. Mater..

[5] Islam M.R., Virag J., Oyen M.L. (2020). Micromechanical poroelastic and viscoelastic properties of ex-vivo soft tissues. J. Biomech..

[6] Wang C.C., Hung C.T., Mow V.C. (2001). An analysis of the effects of depth-dependent aggregate modulus on articular cartilage stress-relaxation behavior in compression. J. Biomech..

[7] Chaudhuri O., Cooper-White J., Janmey P.A., Mooney D.J., Shenoy V.B. (2020). Effects of extracellular matrix viscoelasticity on cellular behaviour. Nature.

[8] Brandl F., Sommer F., Goepferich A. (2007). Rational design of hydrogels for tissue engineering: Impact of physical factors on cell behavior. Biomaterials.

[9] De Leon-Oliva D., Boaru D.L., Perez-Exposito R.E., Fraile-Martinez O., García-Montero C., Diaz R., Bujan J., García-Honduvilla N., Lopez-Gonzalez L., Álvarez-Mon M. (2023). Advanced hydrogel-based strategies for enhanced bone and cartilage regeneration: A comprehensive review. Gels.

[10] Thang N.H., Chien T.B., Cuong D.X. (2023). Polymer-based hydrogels applied in drug delivery: An overview. Gels.

[11] Ahmad Z., Salman S., Khan S.A., Amin A., Rahman Z.U., Al-Ghamdi Y.O., Akhtar K., Bakhsh E.M., Khan S.B. (2022). Versatility of hydrogels: From synthetic strategies, classification, and properties to biomedical applications. Gels.

[12] Chai Q., Jiao Y., Yu X. (2017). Hydrogels for biomedical applications: Their characteristics and the mechanisms behind them. Gels.

[13] Stojkov G., Niyazov Z., Picchioni F., Bose R.K. (2021). Relationship between structure and rheology of hydrogels for various applications. Gels.

[14] Surowiecka A., Strużyna J., Winiarska A., Korzeniowski T. (2022). Hydrogels in burn wound management—A review. Gels.

[15] You J., Liu Z. (2024). Revealing novel insights into the toughening mechanism of double network hydrogels via uniaxial tensile tests. J. Mech. Phys. Solids.

[16] Song R., Wang X., Johnson M., Milne C., Lesniak-Podsiadlo A., Li Y., Lyu J., Li Z., Zhao C., Yang L. (2024). Enhanced strength for double network hydrogel adhesive through cohesion-adhesion balance. Adv. Funct. Mater..

[17] Nonoyama T., Gong J.P. (2015). Double-network hydrogel and its potential biomedical application: A review. Proc. Inst. Mech. Eng. Part H J. Eng. Med..

[18] Gong J.P. (2010). Why are double network hydrogels so tough?. Soft Matter.

[19] Gong J.P., Katsuyama Y., Kurokawa T., Osada Y. (2003). Double-network hydrogels with extremely high mechanical strength. Adv. Mater..

[20] Haque M.A., Kurokawa T., Gong J.P. (2012). Super tough double network hydrogels and their application as biomaterials. Polymer.

[21] Sun J.-Y., Zhao X., Illeperuma W.R., Chaudhuri O., Oh K.H., Mooney D.J., Vlassak J.J., Suo Z. (2012). Highly stretchable and tough hydrogels. Nature.

[22] Li J., Suo Z., Vlassak J.J. (2014). Stiff, strong, and tough hydrogels with good chemical stability. J. Mater. Chem. B.

[23] Agrawal A., Rahbar N., Calvert P.D. (2013). Strong fiber-reinforced hydrogel. Acta Biomater..

[24] Dubey N., Ferreira J.A., Daghrery A., Aytac Z., Malda J., Bhaduri S.B., Bottino M.C. (2020). Highly tunable bioactive fiber-reinforced hydrogel for guided bone regeneration. Acta Biomater..

[25] Yi J., Choe G., Park J., Lee J.Y. (2020). Graphene oxide-incorporated hydrogels for biomedical applications. Polym. J..

[26] Zhang L., Wang Z., Xu C., Li Y., Gao J., Wang W., Liu Y. (2011). High strength graphene oxide/polyvinyl alcohol composite hydrogels. J. Mater. Chem..

[27] Argun A., Can V., Altun U., Okay O. (2014). Nonionic double and triple network hydrogels of high mechanical strength. Macromolecules.

[28] Li X., Tang C., Liu D., Yuan Z., Hung H.C., Luozhong S., Gu W., Wu K., Jiang S. (2021). High-strength and nonfouling zwitterionic triple-network hydrogel in saline environments. Adv. Mater..

[29] Islam M., Oyen M. (2021). Load-relaxation characteristics of chemical and physical hydrogels as soft tissue mimics. Exp. Mech..

[30] Zhao X., Huebsch N., Mooney D.J., Suo Z. (2010). Stress-relaxation behavior in gels with ionic and covalent crosslinks. J. Appl. Phys..

[31] Chaudhuri O., Gu L., Klumpers D., Darnell M., Bencherif S.A., Weaver J.C., Huebsch N., Lee H.-p., Lippens E., Duda G.N. (2016). Hydrogels with tunable stress relaxation regulate stem cell fate and activity. Nat. Mater..

[32] Tang S., Richardson B.M., Anseth K.S. (2021). Dynamic covalent hydrogels as biomaterials to mimic the viscoelasticity of soft tissues. Prog. Mater. Sci..

[33] Islam M.R., Oyen M.L. (2022). Mechanical characterization of hydrogels. The Mechanics of Hydrogels.

[34] Adelnia H., Ensandoost R., Moonshi S.S., Gavgani J.N., Vasafi E.I., Ta H.T. (2022). Freeze/thawed polyvinyl alcohol hydrogels: Present, past and future. Eur. Polym. J..

[35] Hassan C.M., Peppas N.A. (2000). Structure and morphology of freeze/thawed PVA hydrogels. Macromolecules.

[36] Subhash G., Liu Q., Moore D., Ifju P., Haile M. (2011). Concentration dependence of tensile behavior in agarose gel using digital image correlation. Exp. Mech..

[37] Sabzi M., Samadi N., Abbasi F., Mahdavinia G.R., Babaahmadi M. (2017). Bioinspired fully physically cross-linked double network hydrogels with a robust, tough and self-healing structure. Mater. Sci. Eng. C.

[38] Sun X., Luo C., Luo F. (2020). Preparation and properties of self-healable and conductive PVA-agar hydrogel with ultra-high mechanical strength. Eur. Polym. J..

[39] Li H., Wu C.-w., Wang S., Zhang W. (2020). Mechanically strong poly (vinyl alcohol) hydrogel with macropores and high porosity. Mater. Lett..

[40] Weizel A., Distler T., Schneidereit D., Friedrich O., Bräuer L., Paulsen F., Detsch R., Boccaccini A., Budday S., Seitz H. (2020). Complex mechanical behavior of human articular cartilage and hydrogels for cartilage repair. Acta Biomater..

[41] Wheatley B.B., Fischenich K.M., Button K.D., Haut R.C., Donahue T.L.H. (2015). An optimized transversely isotropic, hyper-poro-viscoelastic finite element model of the meniscus to evaluate mechanical degradation following traumatic loading. J. Biomech..

[42] Mattice J.M., Lau A.G., Oyen M.L., Kent R.W. (2006). Spherical indentation load-relaxation of soft biological tissues. J. Mater. Res..

[43] Webber R.E., Creton C., Brown H.R., Gong J.P. (2007). Large strain hysteresis and mullins effect of tough double-network hydrogels. Macromolecules.

[44] Armisen R., Gaiatas F. (2009). Agar. Handbook of Hydrocolloids.

[45] Rivlin R.S., Thomas A.G. (1953). Rupture of rubber. I. Characteristic energy for tearing. J. Polym. Sci..

[46] Tschoegl N.W. (2012). The Phenomenological Theory of Linear Viscoelastic Behavior: An Introduction.

